# Optical modulation of neurotransmission using calcium photocurrents through the ion channel LiGluR

**DOI:** 10.3389/fnmol.2013.00003

**Published:** 2013-03-21

**Authors:** Mercè Izquierdo-Serra, Dirk Trauner, Artur Llobet, Pau Gorostiza

**Affiliations:** ^1^Institute for Bioengineering of Catalonia (IBEC)Barcelona, Spain; ^2^Department of Chemistry and Pharmacology, Ludwig-Maximilians-UniversitätMunich, Germany; ^3^Center for Integrated Protein ScienceMunich, Germany; ^4^Laboratory of Neurobiology, Bellvitge Institute for Biomedical Research (IDIBELL)L’Hospitalet de Llobregat, Spain; ^5^Catalan Institution for Research and Advanced Studies (ICREA)Barcelona, Spain; ^6^Networking Research Center on Bioengineering, Biomaterials and Nanomedicine (CIBER-BBN)Zaragoza, Spain

**Keywords:** optical control, calcium, firing rate, neurotransmission, optogenetics, synaptic transfer function, neural coding

## Abstract

A wide range of light-activated molecules (photoswitches and phototriggers) have been used to the study of computational properties of an isolated neuron by acting pre and postsynaptically. However, new tools are being pursued to elicit a presynaptic calcium influx that triggers the release of neurotransmitters, most of them based in calcium-permeable Channelrhodopsin-2 mutants. Here we describe a method to control exocytosis of synaptic vesicles through the use of a light-gated glutamate receptor (LiGluR), which has recently been demonstrated that supports secretion by means of calcium influx in chromaffin cells. Expression of LiGluR in hippocampal neurons enables reversible control of neurotransmission with light, and allows modulating the firing rate of the postsynaptic neuron with the wavelength of illumination. This method may be useful for the determination of the complex transfer function of individual synapses.

## INTRODUCTION

The investigation of the computational properties of neuronal circuits has required the development of new methods bringing together the morphological properties and physiological activity of neuronal compartments at increasing resolution. As an example, computation of dendritic structures was established with a combination of electrophysiological and imaging techniques (Nikolenko et al., 2007; Branco and Hausser, 2010). More recently, tools to remotely control the activity of neurons with light have been developed (Boyden et al., 2005; Volgraf et al., 2006; Gorostiza and Isacoff, 2008; Deisseroth, 2011). Now, the combination of optogenetic and electrophysiological methods is allowing to test the causality between structure and function: by selectively activating or silencing specific structures like neuronal compartments, one can assess their impact on the function of a neuron, on the dynamics of a circuit, and on the behavior of an organism (Scanziani and Hausser, 2009).

The computational properties of an isolated neuron can be analyzed by postsynaptic activation with compounds caging neurotransmitters, i.e., caged-glutamate (Araya et al., 2006). However, the study of neuronal circuits requires novel tools to gain control of neurotransmitter release by presynaptic terminals. Besides physiological stimulation using patch electrodes or optogenetic approaches, available methods to stimulate exocytosis of synaptic vesicles are restricted to local application of selective Ca^2+^ ionophores (e.g., ionomycin), or Ca^2+^ uncaging (Neher and Zucker, 1993). Dialysis of membrane-impermeant caged Ca^2+^ (e.g., *o*-nitrophenyl-ethylene glycol tetraacetic acid, NP-EGTA) and focused light flashes allow constraining Ca^2+^ release at presynaptic terminals, but this approach is subject to significant intra and inter-experiment variability due to non-homogeneous diffusion of the molecule through the cytoplasm and poor reversibility, thus making this approach virtually non-applicable to a neuronal circuit.

Light-gated ion channels like Channelrhodopsin-2 (ChR2) or the light-gated glutamate receptor (LiGluR) allow optical stimulation with high spatiotemporal resolution. ChR2 has been recently used to map the organization of excitatory connections in the cortex (Petreanu et al., 2009; Mao et al., 2011). LiGluR allows to directly and reversibly control the free concentration of cytoplasmic calcium to trigger regulated exocytosis of large-dense core vesicles in chromaffin cells, showing a comparable efficacy to native voltage-gated calcium channels (VGCCs; Izquierdo-Serra et al., 2012). Calcium-permeable ChR2 mutants have also been identified that should be useful for that purpose (Kleinlogel et al., 2011; Kato et al., 2012). Optical control of calcium influx in neuroendocrine cells opens an experimental window in synapses, as for example, to remotely study the Ca^2+^ dynamics of the process in presynaptic terminals, to gradually trigger neurotransmission and ultimately to modulate the neuronal firing rate by simply changing the illuminating wavelength. All these possibilities would be supported by the ability of LiGluR to efficiently increase submembranous calcium concentration, without requiring the activation of the whole neuron by depolarization.

Here we exploit the wavelength dependence of LiGluR currents (Gorostiza et al., 2007) to achieve a graded control of calcium influx, in order to modulate the secretory rate in chromaffin cells. We also apply this concept to synaptic transmission, by modulating the firing rate of postsynaptic neurons in a wavelength-dependent manner. This technique allows adjusting the firing frequency in a way that is orthogonal to the control of membrane potential (voltage-clamp), and suggests applications to determine experimentally the frequency-dependent transfer function of individual synapses, that could be useful to model neuronal circuits.

## MATERIALS AND METHODS

### CHROMAFFIN CELL CULTURE AND INFECTION

Chromaffin cells were isolated from medulla of bovine adrenal glands by enzymatic treatments (O’Connor et al., 2007). Dissociated chromaffin cells were plated at 2.5 × 10^5^ cells well^-1^ density in Poly-L-lysine treated coverslips. After 1 day, cells were infected using an adenoviral construction carrying the fusion protein GluK2-L439C-eGFP. Amperometry and patch clamp experiments in chromaffin cells were performed after 1–2 days of infection.

### HIPPOCAMPAL NEURONAL CULTURE AND CELL TRANSFECTION

Hippocampal neurons from Albino Sprague-Dawley rats (P1–P4) were isolated and cultured as described previously (Halliwell et al., 1989). Experimental procedures were approved by the Department of Environment from the Generalitat de Catalunya and registered under DMAH #5131. Neurons were plated at 5 × 10^5^ per 12-mm diameter coverslip, previously treated with poly-D-lysine and they were incubated at 37°C and 10% CO_2_. On day 7 to 14 after plating, neurons were transfected with the DNA encoding for GluK2-L439C-eGFP using Lipofectamine 2000 (Invitrogen). Neurons were recorded 2–3 days after transfection.

### CONJUGATION OF MAG PHOTOSWITCH

Maleimide-azobenzene-glutamate (MAG) was synthesized as described (Volgraf et al., 2006) and the concentrated stock (10 mM in dimethyl sulfoxide, DMSO) was stored at -20°C. Before all experiments, cells were incubated in absence of light, for 10 min in a Na^+^-free and low-Ca^2+^ (0.5 mM) solution with 10–100 μM of MAG (DMSO final concentration <1%) and 0.3 mg mL^-1^ concanavalin A, to block GluK2 desensitization (Gorostiza et al., 2007).

### CARBON MICROFIBER AMPEROMETRY

Catecholamine release was detected using homemade polyethylene-insulated carbon fiber electrodes of 12-μm diameter (Chow and von Rüden, 1995; Dernick et al., 2005). Amperometric electrodes were first tested in a solution containing 5 mM ferricyanide in 0.1 M KCl and pH 6.8. Electrodes displaying a current between 1 and 10 nA at a holding potential of +700 mV were selected, and their integrity was verified by voltammetry (Schulte and Chow, 1996). Only electrodes showing a symmetric oxidation/reduction current response to a symmetric ramp from +700 to -300 mV (scan rate 100 mV s^-1^) were used to measure exocytosis in chromaffin cells. When necessary, electrodes were freshly cut with a scalpel on a glass surface, and were used for further experiments if the basal current was between 10 and 20 pA (at holding potential +700 mV) in the bath solution. Amperometric current was recorded by applying a holding voltage of +700 mV with an EPC-10 amplifier (HEKA) controlled with Patch Master (HEKA). The sampling rate was 100 kHz and current was filtered with a Bessel Filter set at 30 kHz. After data acquisition, traces were digitally filtered at 1 kHz.

### CURRENT-CLAMP RECORDINGS

Recordings of current-clamp under whole-cell configuration were done using an EPC-10 amplifier and Patch Master. Pipettes were pulled from borosilicate glass tubing (Harvard Apparatus) with P-97 puller from Sutter Instruments, with a typical resistance of 3–6 MΩ. Voltage was acquired at a sampling rate of 25 kHz and filtered with a Bessel Filter set at 30 kHz. Membrane voltage was held at -70 mV before switching to current-clamp mode, and the injected current was corrected if basal membrane voltage drifted above -60 mV.

### VOLTAGE-CLAMP RECORDINGS

Borosilicate glass pipettes were pulled with a typical resistance of 2–4 MΩ. Voltage-clamp recordings under whole-cell configuration were done using an EPC-10 amplifier and the Patch Master. Cell membrane was clamp at a holding potential (*V*_h_) of -80 mV and current was acquired at a sampling rate of 20 kHz. Before each train of light stimulus a hyperpolarization of -90 mV was applied to later allow leak subtraction to ion currents. For the current density–voltage relationship, the following pulse protocol was used: *V*_h_ = -80 mV, test pulses of 20 ms at steps between -100 and +50 mV (10 mV increment), and P/5 leak subtraction protocol. First, neurons were bathed in a physiological solution with 1 μM of tetrodotoxin (TTX, Ascent Scientific). Then, to quantify the VGCC inhibition, the toxin cocktail was directly added to the bath. Toxins get the maximal VGCC block after 15 min, when current density–voltage relationship was measured again from the same neuron.

### ILLUMINATION

Illumination was applied to the entire focused field, using a TILL Photonics Polychrome V monochromator through the side port of an IX70 inverted microscope (Olympus) and with a UApo/340, 40×/1.35 objective. Shutter and wavelength were controlled trough EtherNet-COM-1 connection to PC, using TILL Photonics Polychrome V Control (PolyCon) software. The light power measured with light meter model Newport 1916-C placed next to the objective was 0.9 mW mm^-2^ at 380 nm and 1.7 mW mm^-2^ at 500 nm.

### DATA ANALYSIS

All analysis was done with IgorPro from Wavemetrics. For amperometric spike and action potential (AP) detection and parameter analysis (Mosharov and Sulzer, 2005) *Igor Procedures Quanta Analysis* macro from Eugene Mosharov laboratory was used (http://www.sulzerlab.org). Data was exported to Matlab to calculate secretion and firing rate with a custom made macro that calculates the number of events per second in 20 ms windows. Statistical tests were done with Matlab. For all groups of data we applied a non-parametric multiple comparison test (Kruskal–Wallis) and a multicompare least significant difference (LSD) test. All data are expressed as mean ± SEM (standard error of the mean, calculated over the number of *N*).

### SOLUTIONS

Composition of physiological bath solution (in mM): 140 NaCl, 2.5 KCl, 1 MgCl_2_, 10 HEPES 4-(2-hydroxyethyl)piperazine-1-ethanesulfonic acid, 10 glucose, 2.5 CaCl_2_ at pH 7.42 and 300 mOsm kg^-1^. The composition of pipette solution for voltage-clamp was (in mM): 120 Cesium methanesulfonate, 10 TEA-Cl (tetraethylammonium chloride), 20 HEPES, 3 Na_2_ ATP, 1 NaGTP, and 0.5 EGTA, pH 7.2 and 290 mOsm kg^-1^. In current-clamp recordings, the pipette solution contained (in mM): 130 KCl, 5 MgCl, 3 NA_2_ATP, 1 NA_2_GTP, 20 HEPES, 0.5 EGTA, pH 7.2 and 290 mOsm kg^-1^. In the indicated experiments, the following cocktail of toxins was added to the bath solution: 100 nM ω-agatoxin IVA (and 1 μM ω-conotoxin GVIA from Alomone Labs and 10 μM nifedipine. When indicated, TTX (Ascent Scientific) was added to the bath solution at 1 μM.

All reagents were obtained from Sigma unless otherwise specified.

## RESULTS

### MAG OPTICALLY GEARS NEUROSECRETION

**FIGURE 1:**
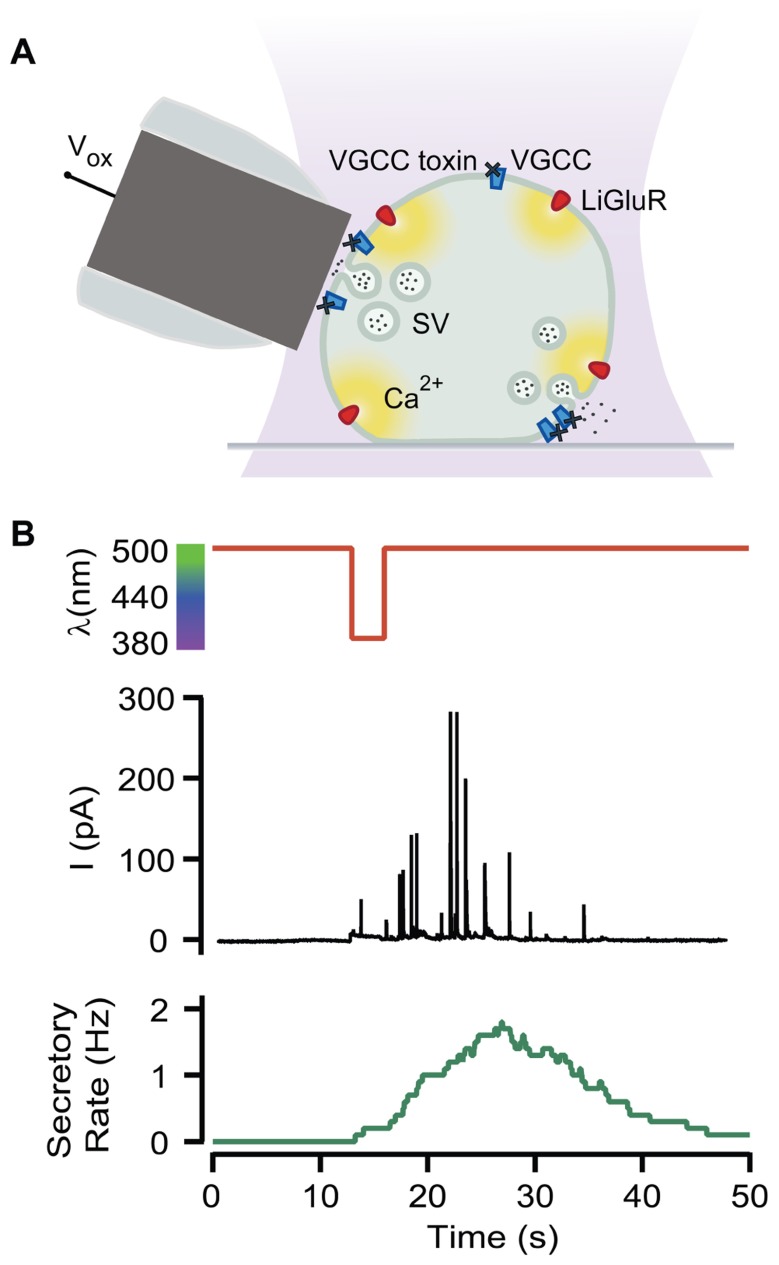
**Optical triggering of neurosecretion**. **(A)** Diagram of the experimental approach. Secretion upon light stimuli was detected by amperometry in LiGluR(+) chromaffin cells while VGCC where blocked by toxins (*V*_ox_, oxidation voltage; SV, secretory vesicle). **(B)** Amperometric current recording (black plot) from a single LiGluR(+) cell in response to 5 s, 380 nm light pulse (red plot), and the secretory rate (green plot).

**FIGURE 2:**
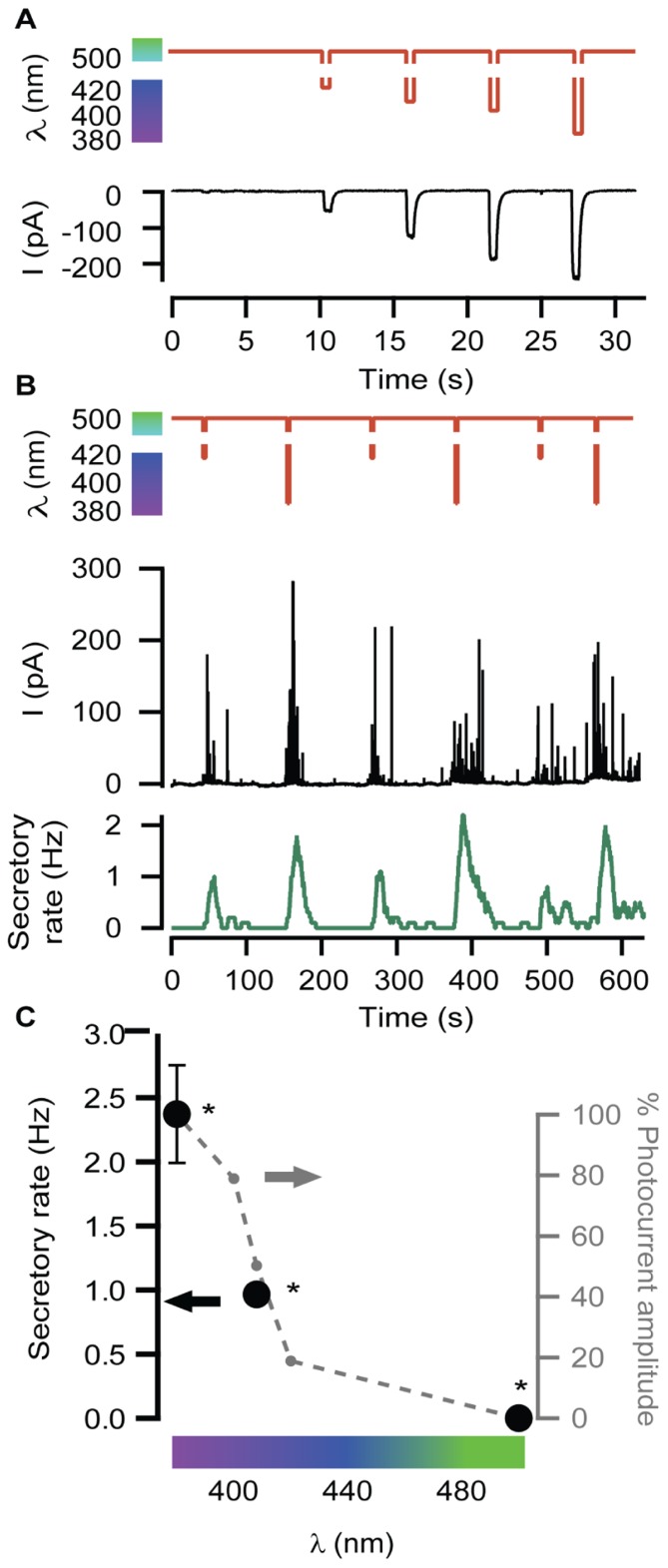
**Modulation of secretory rate**. **(A)** LiGluR current amplitude can be adjusted with the illumination wavelength: whole-cell patch clamp current responses (black) to light pulses between 500 nm and different UV wavelengths (sequence of pulses of 420, 408, 400, and 380 nm, red plot). **(B)** The wavelength of light stimuli (red plot) allows adjusting the size of the burst of amperometric spikes (black plot) and thus the maximum secretory rate in each burst (green plot). The exocytosis rate always returns to 0 under 500 nm light, and can be driven reproducibly at 1 or 2 Hz by stepping to 408 or 380 nm, respectively. **(C)** Plot of the averaged secretory rate in response to 380, 408, and 500 nm light (•, left *y*-axis, *N* = 4 cells) measured by amperometry. In gray, photocurrent amplitude dependence on wavelength illumination in LiGluR(+) chromaffin cells measured by whole-cell voltage-clamp (-•-, right *y*-axis, *N* = 4 cells). All pairs are significantly different (*p* = 0.12) and were compared using a non-parametric multiple comparison test (Kruskal–Wallis) and a multicompare LSD test. Error bars indicate ±SEM.

Neurosecretion can be triggered by light in bovine chromaffin cells expressing LiGluR [LiGluR(+)], due to the calcium photocurrent (Izquierdo-Serra et al., 2012). Light-triggered exocytic events are detected by amperometry (**Figure 1A**) or by whole-cell membrane capacitance recordings, while keeping the endogenous VGCCs blocked to avoid calcium entry due to depolarization. In order to inhibit the three types of VGCCs expressed in bovine chromaffin cells: Ca_v_1, Ca_v_2.2, and Ca_v_2.1, cells were bathed into a physiological solution containing nifedipine, ω-conotoxin GVIA, and ω-agatoxin IVA (Garcia et al., 2006). **Figure 1B** shows that a 5-s illumination pulse at 380 nm (red trace), evokes catecholamine release detected as spikes in the amperometric current recording (black trace). The secretory rate (green trace) can be calculated from the amperometric trace and rises up to 2 Hz upon 380 nm light stimulation, gradually decaying once light is switched back to 500 nm. The 380 nm wavelength opens maximally LiGluR and thus triggers a maximal photoinduced secretory rate (Izquierdo-Serra et al., 2012). To gear optical control of neurosecretion, we took advantage of the graded behavior that the MAG photoswitch elicits on LiGluR channels (Gorostiza et al., 2007). As indicated in **Figure 2A**, the amplitude of cationic currents flowing through LiGluR channels is proportional to the illumination wavelength between 420 and 380 nm, which we used to finely modulate secretion.

In **Figure 2B**, secretion was repeatedly triggered with light as in **Figure 1**, but illuminating alternately at wavelengths producing variable Ca^2+^ currents, which account for roughly 10% of the measured cationic current (Izquierdo-Serra et al., 2012). The result is illustrated by the amperometric response (black trace) of a single LiGluR(+) cell to this wavelength range, and the calculated frequency (green trace). As can be seen in **Figure 2B**, secretion was stopped at 500 nm and it could be driven at low (~0.1–0.8 Hz) or high secretory rate (2–4 Hz) depending on whether cells were illuminated at 408 or 380 nm, respectively. The off, low and high rates of neurosecretion were reproducibly alternated.

The plot on **Figure 2C** summarizes the averaged values obtained from amperometric experiments in several individual chromaffin cells (black). It points out that the light-triggered secretory rate can be directly regulated with the illumination wavelength as a consequence of the control of the photocurrent amplitude (**Figures 2A,C** in gray).

### LiGluR-MEDIATED Ca^2+^ INFLUX IN THE PRESYNAPTIC NEURON-INDUCED POSTSYNAPTIC ACTION POTENTIALS

Having shown that exocytosis can be triggered and modulated with light by means of LiGluR-mediated Ca^2+^ influx in chromaffin cells, we aimed at extending such control to neurotransmitter release at chemical synapses.

**FIGURE 3:**
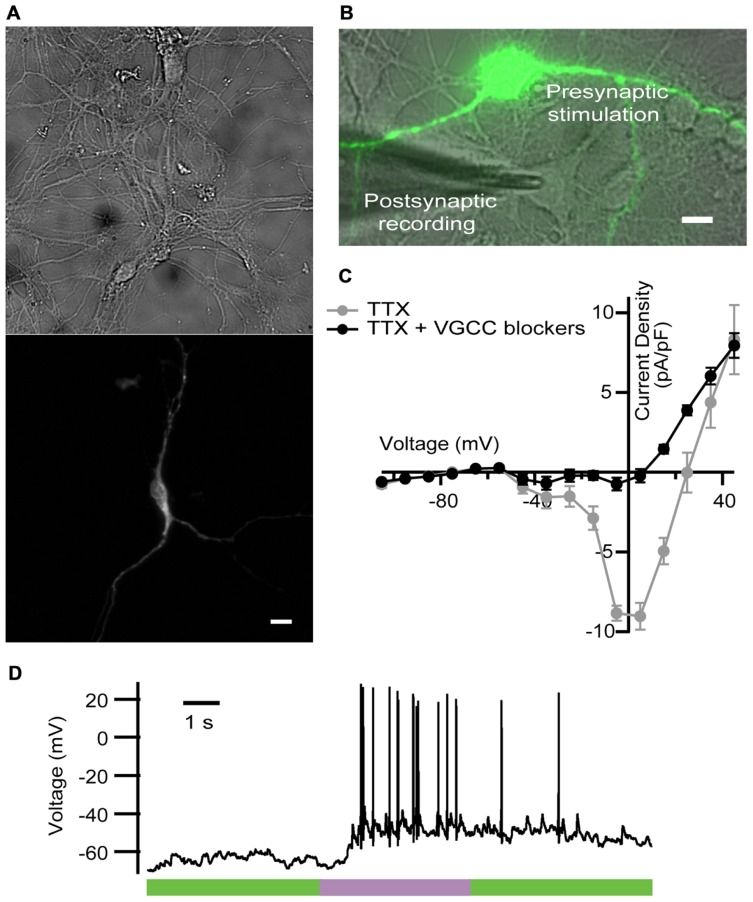
**LiGluR-mediated Ca^2+^ influx in the presynaptic neuron-induced postsynaptic AP**. **(A)** LiGluR is expressed in the neuron soma and in all processes, as indicated by fluorescence from green fluorescent protein (GFP) fused to GluK2-L439C under a cytomegalovirus (CMV) promoter (top: bright field image; bottom: GFP). Scale bar, 10 μm. **(B)** Image of the presynaptic LiGluR(+) neuron (green fluorescence), and the glass pipette recording from a LiGluR(-) postsynaptic neuron in contact with the green one. Scale bar, 10 μm. **(C)** Quantification of the voltage-gated Ca^2+^ current inhibition by the toxin cocktail against VGCC in hippocampal neurons. Current density–voltage relationship in absence (gray) and in presence of the toxin cocktail (black) measured in the same neuron. Isolation of the Ca^2+^ current was obtained by blocking voltage-gated Na^+^ channels with 1 μM of TTX in the physiological bath solution, where up to 91.6% of the VGCC currents were inhibited (*N* = 4 cells with cell capacitance between 14.5 and 26.5 pF). Error bars indicate ±SEM. **(D)** Representative voltage recording from postsynaptic neuron in the presence of VGCC blockers, purple bar indicates 2 s, 380 nm illumination.

For that purpose, we expressed LiGluR in rat cultured hippocampal neurons, and stimulated them with light while blocking VGCC-mediated Ca^2+^ currents as done in the previous experiments in chromaffin cells. LiGluR expression was observed in the soma and in all processes (**Figure 3A**). Using whole-cell patch clamp in the current-clamp mode, we recorded the membrane potential of non-transfected, LiGluR(-) neuron in the vicinity of a LiGluR(+) neuron and (post)synaptically connected to it (**Figure 3B**). We aimed to record APs generated at the LiGluR(-) postsynaptic neuron, as a consequence of the neurotransmitter release from the LiGluR(+) stimulated by light. To validate this assay, two control experiments were done. First, in order to rule out that the recorded neuron was expressing any LiGluR, we always verified the absence of voltage-clamped current responses to UV stimulation, discarding the cell when it responded to light. In other control experiments, recording from LiGluR(+) neurons we assessed the efficacy of VGCC block by comparing the current density–voltage relationship before and after adding the toxin cocktail. Before the cocktail the current density–voltage curve presents one peak at -30 mV and one at 0 mV corresponding to the activation of low- and high-threshold Ca^2+^ channels, respectively. Currents were reduced to less than 10% at 0 mV (due to Ca^2+^ channels resistant to low ω-agatoxin IVA concentration, 100 nM; Garcia et al., 2006), and 50% at 30 mV with the toxins, and were completely blocked with Cd^2+^, which corroborates that the remaining currents were due to the presence of Ca^2+^ channels (**Figure 3C**). The total charge mobilized by VGCCs and LiGluR can be calculated taking into account that VGCC currents inactivate and that non-desensitizing LiGluR currents have a 10% calcium component (Izquierdo-Serra et al., 2012). Thus, VGCCs (including low-threshold) give rise to a calcium charge of 9 ± 4 pC (*N* = 21) during 2 s stimulation pulses, which is 10-fold smaller than that of LiGluR during the same time (90 ± 20 pC, *N* = 11). Under these conditions, and in the presence of physiological bath solution, LiGluR(+) neurons can still be depolarized with light (due to the cationic influx), but APs traveling along axons produce a small Ca^2+^ entry through VGCCs in comparison to LiGluR. Therefore, the main pathway for Ca^2+^ influx is controlled by illumination.

We thus proceeded with the experiment described in **Figure 3B**. A representative trace is shown in **Figure 3D**. At the beginning of the recording neurons showed a stable membrane potential or few spontaneous AP. During the light pulse of 2 s at 380 nm, a train of APs was generated, and when the light was switched to 500 nm APs were gradually reduced.

We observed a delay between the beginning of the stimulus and the generation of the first AP (see **Figure 3D**), which was longer (0.74 ± 0.09 s, *N* = 4 neurons stimulated at 380 nm) that the value that has been reported in hippocampal neurons (100 ms). As discussed in detail for chromaffin cells (Izquierdo-Serra et al., 2012), such delay may arise from poor coupling between LiGluR channels and synaptic vesicles, compared to VGCCs. In these experiments, the calculated firing rate on the current-clamped postsynaptic neuron reversibly switched between 0 Hz under 500 nm and a maximum rate under 380 nm illumination. Maximum frequency ranged from 1.3 to 10 Hz, and was 6 ± 2 Hz (*N* = 4 neurons) on average. Altogether, these results indicate that the LiGluR-mediated Ca^2+^ influx in the presynaptic neuron triggers regulated exocytosis of neurotransmitter containing vesicles, thus supporting the generation of postsynaptic APs.

### LIGHT-GATED SECRETION AT PRESYNAPTIC TERMINALS SUPPORTS COLOR-MODULATED NEUROTRANSMISSION AND CONTROLS POSTSYNAPTIC FIRING RATE

**FIGURE 4:**
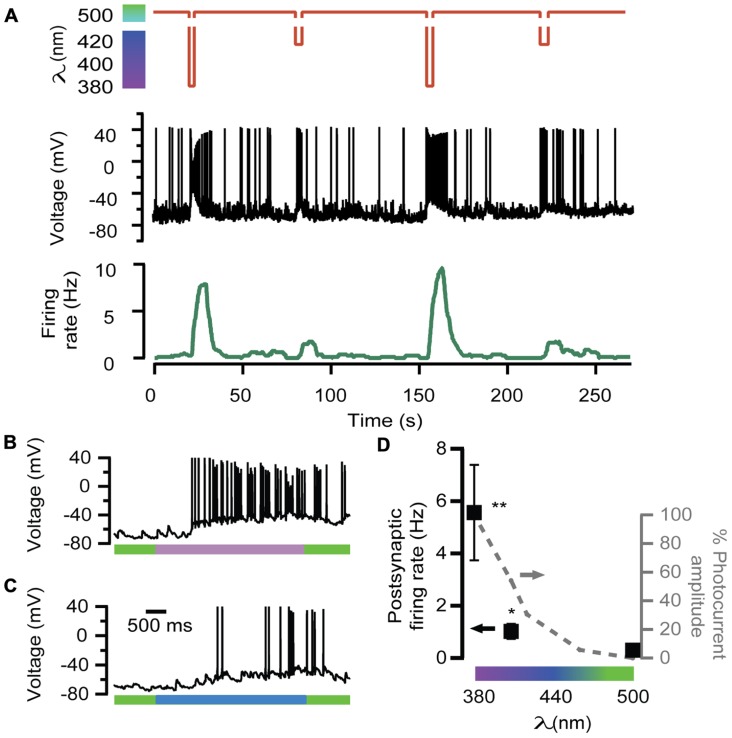
**Wavelength modulation of postsynaptic firing rate in hippocampal neurons**. **(A)** Alternated illumination at 380 and 408 nm was spaced by a 45-s period under 500 nm to recover resting membrane voltage (red trace). Voltage recording of the APs on the postsynaptic neuron (black trace) and the calculated firing rate (green trace). Note that AP bursts are associated with light stimulation pulses, and the maximum values of firing rate depend on wavelength illumination. **(B,C)** Two voltage recordings from the same postsynaptic neuron expanded from **(A)**, during illumination at 380 nm (purple bar, up) or 408 nm (blue bar, down). **(D)** Plot of the averaged firing rate from postsynaptic LiGluR(-) neurons, in response to 380, 408, and 500 nm light pulses (•, left *y*-axis, *N* = 4 cells) and photocurrent amplitude of the presynaptic neuron at the same wavelengths measured in LiGluR(+) hippocampal neurons by whole-cell voltage-clamp, following the same protocol described in **Figure 2A** (-•-, right *y*-axis, *N* = 4 cells). **Significantly different from 500 nm with *p* < 0.05; *significantly different from 500 nm with *p* < 0.1. All pairs were compared using a non-parametric multiple comparison test (Kruskal–Wallis) and a multicompare LSD test. Error bars indicate ±SEM.

In order to further test the ability of LiGluR to adjust the excitatory input, neurotransmission was evoked by 380, 408, and 500 nm light wavelengths, which geared exocytosis at different secretory rates in chromaffin cells (**Figure 2A**). The amount of APs generated by 408 nm illumination was lower than by 380 nm (**Figures 4C,B**, respectively). The postsynaptic firing rate calculated was 2 and 10 Hz for 408 and 380 nm, respectively, and it changed reversibly and reproducibly according to light wavelength (**Figure 4A**).

The average of various responses is represented in the plot of **Figure 4D**, which shows that neurotransmission, assayed through the firing rate, was modulated in proportion to the color of illumination.

## DISCUSSION

Light-gated glutamate receptor allows controlling secretion with light in chromaffin cells under physiological extracellular Ca^2+^ concentrations, as demonstrated by amperometry and membrane capacitance recordings (Izquierdo-Serra et al., 2012). Interestingly, LiGluR allows a color control of secretory events by adjusting the illumination wavelength between 380 and 500 nm (**Figure 2B**). This maneuver sets the fraction of open channels (*cis*-MAG) and thus the total Ca^2+^ influx. At each wavelength, the relative abundance of *cis*- and *trans*-MAG results from the relative optical absorption of each isomer because inter conversion between them occurs until a photo stationary state is reached (Fischer, 1967; Gorostiza et al., 2007). In chromaffin cells the control of secretory rate with light wavelength was limited to three “gears”: no secretion, medium and maximum secretion (**Figure 2B**). Although gating of LiGluR can be finely adjusted by the color of illumination, which would theoretically expand the number of “gears,” our results show that in practice such tuning of neurosecretion is not viable. A plausible explanation resides on the fourth power relationship between neurotransmitter release and extracellular calcium (Dodge and Rahamimoff, 1967). This limitation, however, does not preclude the possibility of using LiGluR to perform a remote color-encoded manipulation of firing rate in neural circuits, which is reminiscent of color-encoding of neuronal identity with the optical probe Brainbow (Livet et al., 2007).

Chromaffin cells constitute a well-established model to study the exocytic processes that occur in presynaptic terminals. We have described conditions under which LiGluR behaves as a light-gated Ca^2+^ channel that mediates a sufficiently large Ca^2+^ influx to support regulated exocytosis (Izquierdo-Serra et al., 2012). Furthermore, the exocytic rate can be geared with the wavelength of illumination (**Figure 2**) and this property can be translated to chemical synapses by utilizing LiGluR as a presynaptic channel (**Figure 4**). Although other light-gated, Ca^2+^-permeable channels like ChR2 can support neurotransmitter release by depolarizing individual synapses and activating VGCCs (Petreanu et al., 2009), their application could be improved by directly triggering exocytosis in a wavelength-dependent manner, a possibility that is limited in practice by the reduced conductivity and Ca^2+^ permeability of ChR2 (Miesenbock, 2009; Li et al., 2012). On the other hand, Ca^2+^ uncaging to the cytoplasm (Neher and Zucker, 1993) is poorly reversible and less physiologically relevant than Ca^2+^ entry through the membrane (Denker and Rizzoli, 2010), and cannot be genetically targeted to the cells of interest. Thus, LiGluR stands as the best available method to remotely and reversibly trigger Ca^2+^-regulated exocytosis in neurons. However, the delay observed between illumination and the first AP points to a weak coupling between LiGluR and synaptic vesicles that leads to slow Ca^2+^ buildup (Izquierdo-Serra et al., 2012), and/or to the possibility that light-stimulated neurons and recorded neurons in the preparation are connected polysynaptically or indirectly through the network, and thus the observed optical modulation of neurotransmission probably reflects overall changes in the excitability of the network. Future improvements include LiGluR constructs to enhance presynaptic targeting and coupling to synaptic vesicles, and applying them to preparations where LiGluR(+) neurons establish monosynaptic connections. In these conditions, a systematic correlation between photocurrent amplitude and firing rate could be carried out by paired patch clamp recordings of the presynaptic LiGluR(+) and postsynaptic LiGluR(-) neurons. In that way, the wavelength of light may provide analog control on the synaptic strength, without resorting to changes in the extracellular Ca^2+^ concentration, which affect all synapses in the preparation. Similar progress could be achieved with recently discovered ChR2 variants displaying high permeability to Ca^2+^ (Kleinlogel et al., 2011), or with rationally designed mutants based on ChR2 structure (Kato et al., 2012), although LiGluR has been reported to provide larger photocurrent, shorter illumination required to fire APs, and lack of desensitization (Szobota et al., 2007). LiGluR also has a larger conductivity and is five times ($P_{Ca}^{2+}/P_{Na}=1.2$; Egebjerg and Heinemann, 1993) more Ca^2+^-permeable than CatCh ($P_{Ca}^{2+}/P_{Na}=0.24$; Kleinlogel et al., 2011). In addition, the method based on LiGluR can benefit from synthetic variants of MAG switches with tuned optical properties, as it has been shown for other photoswitches (Mourot et al., 2011).

Understanding the full computational properties of a neuron connected in a circuit requires the characterization of individual synapses by means of the complex transfer function (i.e., with the explicit frequency dependence and not just a measure of “synaptic strength” at low frequencies; Buchanan, 1992; Buchanan et al., 1992; Markram et al., 1998; Abbott and Regehr, 2004). This could be achieved with a large calcium photocurrent channel, by recording the postsynaptic firing pattern in response to a wavelength ramp. Determining the complex transfer function of all synapses in a neuron would allow integrating the overall neuronal transfer function, and lead to cracking neural rate coding.

## Conflict of Interest Statement

The authors declare that the research was conducted in the absence of any commercial or financial relationships that could be construed as a potential conflict of interest.
